# Child health screening program in French nursery schools: Results and related socioeconomic factors

**DOI:** 10.3389/fped.2023.1167539

**Published:** 2023-05-04

**Authors:** Karen Milcent, Malamine Gassama, Marie-Noëlle Dufourg, Xavier Thierry, Marie-Aline Charles, Corinne Bois

**Affiliations:** ^1^ ELFE Joint Unit INED-INSERM-EFS, Paris, France; ^2^ Centre for Research in Epidemiology and Statistics (CRESS), INSERM, INRAE, Universite de Paris, Paris, France; ^3^ Service Départemental de Protection Maternelle et Infantile, Conseil Départemental de l’Essonne, Evry, France

**Keywords:** child, primary healthcare, social inequalities, early childhood disclose, general health

## Abstract

**Objectives:**

The study aims to describe the output of routine health screening performed in French nursery schools by the maternal and child health services among children aged 3–4 years and to quantify the level of early socioeconomic health disparities.

**Methods:**

In 30 participating *départements*, data on screening for vision and hearing impairments, overweight and thinness, dental health, language, psychomotor development, and immunizations were collected for children born on specific dates in 2011 and enrolled in nursery school in 2014–2016. Information was collected on the children, their socioeconomic characteristics and on the school attended. Odds of abnormal screening results were compared for each socioeconomic factor by logistic regressions adjusted for age, sex, prematurity and bilingualism.

**Results:**

Among the 9,939 children screened, prevalence of disorders was 12.3% for vision, 10.9% for hearing, 10.4% for overweight, 7.3% for untreated caries, 14.2% for language and 6.6% for psychomotricity. Newly detected visual disorders were more frequent in disadvantaged areas. Children with unemployed parents were three time more likely to have untreated caries and twice as likely to present language or psychomotor impairments; 52% were referred to a health professional following screening compared to 39% of children with employed parents. Except for children in disadvantaged areas, vaccine coverage was lower among disadvantaged groups.

**Conclusion:**

The prevalences of impairments, which are higher among disadvantaged children, highlight the potential preventive impact of systematic screening under the comprehensive maternal and child healthcare program. These results are important to quantify early socioeconomic inequalities in a Western country known for its generous social welfare system. A more holistic approach to child health is needed with a coherent system involving families and aligning primary care, local child health professionals, general practitioners, and specialists. Further results are needed to evaluate its impact on later child development and health.

## Introduction

To improve overall child health through health promotion in schools, the French maternal and child health services (*Protection Maternelle et Infantile, PMI*) provide a nursery school intervention addressing multiple aspects of health. PMI services are required to carry out a mandatory and comprehensive medical examination on all children aged between 3 and 4. The nursery school is an ideal setting for promoting child development and health. First, the early childhood period is considered to be crucial for later development (1, 2); the positive impacts of early child interventions in later life, especially for disadvantaged families, have been shown (3–5). Second, almost all children in France attend 3 years of nursery school from ages 3 to 6.

PMI services are run by local authorities at *département* level and Preschool Child Health Screening (PCHS) coverage varies across the country (6). While PMI physicians and nurses are required to perform PCHS in all schools within their *départment*, recent publications have shown that just 70% of nursery school children are examined in at least one of the recommended screenings (6, 7). Social inequalities in health in 4-year-old children have been described in some specific *départements* (8), but nationwide PCHS health data are not compiled on a systematic basis and social indicators for this program are not available.

A joint survey, called Elfe-PMI, conducted by the Elfe national child cohort (9) and volunteer PMI services in 30 *départements,* provides an overview of child health at ages 3–4 years, as assessed through the PCHS, and its variations across social indicators. Results on variations in PCHS data across *départements* have already been published (6).

To inform about overall health and development at this age, the present analysis of the Elfe-PMI survey 1) describes the output of a multi-component PCHS of 3–4 year-old children performed by PMI services in France and 2) documents the level of socioeconomic heath disparities at this age.

## Population and methods

### Participants, setting and study design

The Elfe team contacted all PMI services in metropolitan France and 30 out of 96 agreed to participate in the Elfe-PMI survey.

The Elfe cohort follows children born in mainland France on 25 specific days in 2011 spread across four seasonal waves (9). Consequently, all children born in 2011 on the same specific days and enrolled in nursery school during the Elfe-PMI survey period from 2014 to 2016 in one of the 30 participating *départements* were eligible, whether or not they belonged to the Elfe cohort. All parents of eligible children were informed about the study by a letter sent to their nursery school by the PMI service, and a letter from the Elfe unit for children in the Elfe cohort living in the participating *départements*. Using a refusal form, parents could refuse to include their child in the survey without affecting their child's participation in the PHCS.

The Advisory Committee on the Processing of Information in the Field of Health Research (CCTIRS) approved the research. The National Data Protection Authority (CNIL) authorized the processing of survey data (DR-2014-524).

### Child health data

PMI physicians or nurses recorded screening results *via* questionnaires. A complete PHCS includes: vision, hearing, and dental screening; measures of height and weight; immunization history; tests of language and psychomotor development. Parents were invited to attend the examination. When disorders were found, referrals to the child's physician or specific therapists were given in writing directly to the parents if present, or *via* the school communication channels. The number of tests and the screening methods used corresponded to the standard practice of each PMI service. The results of the different screening tests were defined according to the following indicators:

### Vision screening

Vision tests included checking for strabismus, stereopsis disorder and visual acuity in both eyes and each eye at a distance of 2.5 meters using a chart with letters or pictures. For children not previously wearing glasses or an eye patch, a positive screening result for visual impairments was recorded in case of strabismus, stereopsis disorder, visual acuity below 7/10 or a difference between the two eyes of more than 2/10 (10).

### Hearing screening

The hearing screening procedure included a whispered voice test (behind the child at arm's length, the examiner whispers names of objects that the child has to point to on a picture board) or a pure tone audiometry test (500, 1,000, 2,000 and 4,000 hertz frequencies). Screening for hearing problems was considered as positive for one or two affected ears, in case of more than one mistake in the whispered voice test or an inability to hear sounds below 30 decibels.

### Overweight and thinness screening

Body mass index (BMI) was calculated from standing height and weight (kg/m²). Overweight, obesity and thinness were defined according the International Obesity Task Force (IOTF) thresholds for age and sex (11).

### Dental screening

Untreated dental caries were considered as a positive dental screening result.

### Speech and language impairment screening

For expressive and receptive language assessment, three skills were tested: ability to form a sentence, to speak intelligibly, to tell a story (with or without a picture). The examiner recorded whether each one was always, sometimes or never met. Language level was categorized into: no speech language impairment (SLI) if three criteria were always met or two were always met and another one was sometimes met, SLI in the others cases.

### Psychomotor impairment screening

Neuropsychomotor milestones were assessed by six skills, and whether or not the child could perform the associated tasks. We categorized skills into three functional areas: gross motricity, fine motricity or praxis, and perceptual organization, each including 2 items. Gross motor *skills* were forward jump and balance on one leg *for three seconds; fine motor* skills *consisted in drawing a man and showing partial autonomy for dressing; and perceptual skills in recognizing three colors and spatial orientation (find the classroom and the coat rack).* Each functional area was considered satisfactory if at least one of the two respective tasks was performed. If this was not the case, an impairment of psychomotor functions was recorded.

### Vaccination coverage

Diphtheria-Tetanus-Poliomyelitis (DTP), pertussis, pneumococcal, meningococcal C, measles, mumps and rubella (MMR) and hepatitis B vaccine coverage were defined according to the current French immunization schedule.

### Referrals to health professionals

Following the PCHS, children were referred, if necessary, to a pediatrician or specific therapist, depending on the type of disorder detected.

### Socioeconomic and early life factors

Factors known for their association with developmental impairments (12) were recorded during the examination. They included child-related factors: age, sex, preterm birth; school-related factors: schooled in a disadvantaged area that receives additional educational resources (*zones d'éducation prioritaire, ZEP*); family factors: second language other than French spoken at home, single-parent home, mother and/or father unemployed; for employed parents, occupational category (OC): higher-level, intermediate, clerical/sales worker, manual worker (when both parents were employed, the higher OC was used).

### Statistical analysis

Univariate description of the children and of screening results is given with percentages and 95% confidence intervals (95% CI). Impairments detected during screening were calculated for all children and by age, sex, prematurity status, bilingualism and socioeconomic indicators and then compared for these indicators by a Chi² test. In the subgroup of children without missing information on covariates, odds of abnormal screening by single-parent home, parental employment status, parental OC and schooling in a ZEP were tested by logistic regressions adjusted for age, sex, prematurity, bilingualism. An additional adjustment for hearing impairment was performed for SLI. In a sensitivity analysis we also performed a multivariate analysis on the whole sample with a missing value category for all covariates.

## Results

### Sample and participant characteristics

An estimated 97.7% of 4-year-old children were enrolled in nursery school during the Elfe-PMI study period (2014–2016) (13). Therefore, out of the 18,071 children born in 2011 on one of the 25 days of inclusion in the Elfe cohort, an estimated 17,600 attending nursery schools in the 30 participating *départements* were eligible for the study. As the PMI covers 80% of children screened *via* the PCHS in these *départements* (4), our target population totaled around 14,000 children. Among these, 10,447 questionnaires were completed, 1,198 parents refused to participate, and we assumed that the others were screened but not included. After excluding duplicates and ill-identified questionnaires, data were eventually obtained for 9,939 children. Participant characteristics are shown in Table 1.

**Table 1:** Children, school and family characteristics (*n* = 9939).

	Missing (*N*)	% (*N*) or mean SD
**Age** (months)	239	48.7 ± 5.1
**Gender**	2	
Female		49.3 (4,894)
Male		50.7 (5,043)
**Preterm**	2,062	6.9 (542)
**Nursery school year**	77	
First		39.6 (3,903)
Second		60.3 (5,948)
Third		0.1 (11)
**Public School**	2,172	88.3 (6,860)
**Disadvantaged area (ZEP)**	255	12.4 (1,205)
**Bilingualism**	1,040	26.9 (2,391)
**Single Parent**	1,589	10.4 (866)
**Employed Mother**	1,926	69.6 (5,581)
**Employed Father**	2,304	90.8 (6,936)
**Mother's occupational category**	3,264	
Higher-level		18.3 (1,223)
Intermediate		21.1 (1,411)
Self-employed		6.1 (410)
Clerical/sales worker		49.7 (3,320)
Manual worker		4.7 (311)
**Father's occupational category**	2,504	** **
Higher-level		19.8 (1,471)
Intermediate		12.9 (956)
Self-employed		15 (1,115)
Clerical/sales worker		33.8 (2,516)
Manual worker		18.5 (1,377)
**Parental presence during screenings**	280	58.5 (5,653)

ZEP, Zone d’éducation prioritaire.

### Multi-component child health screening

#### Vision screening

Vision screening was performed on 9,417 children (94.7%) and impairments were detected in 12.3% of children who did not wear glasses (Table 2). A total of 806 children (8.1%) were already wearing glasses. Newly detected visual impairments were thus more frequent than those already corrected with glasses. Among the children schooled in a ZEP, 8.6% already wore glasses or had already been screened by an ophthalmologist vs. 10.5% of the other children (*p* = 0.04). Risk factors for newly screened visual impairment in the multivariate analyses were schooling in a ZEP and clerical or manual worker parental OC (Table 3).

**Table 2:** Prevalences of screened abnormalities by selected child characteristics and socioeconomic factors.

	Screening impairment %							Referrals to health professionals %
	Visual impairment^*^	Hearing impairment	Overweight	Thinness	Untreated caries	Language Impairment	Psychomotor impairment	
**Overall** % [IC 95%]	12.3 [11.6–13.0]	10.9 [10.3–11.6]	10.4 [9.7–11.0]	4.6 [4.2–5.1]	7.3 [6.7–8.0]	14.2 [13.5–14.9]	6.6 [6.1–7.1]	41.3 [40.4–42.3]
**Child factors**								
**Age**								
<4 years	13.2	12.5	8.7	4.9	6.2	17.3	10.1	42.1
≥4 years	11.7^a^	10.0^c^	11.3^c^	4.5	8.0^b^	12.2^c^	4.4^c^	40.8
**Gender**								
Female	11.8	9.7	11.7	5.0	6.8	10.5	5.5	40.4
Male	12.6	12.2^c^	9.1^c^	4.3	7.8	17.8^c^	7.7^c^	47.8^c^
**Preterm**								
<37 weeks	13.0	11.5	9.5	5.5	10.2	20.9	10.9	47.8
≥37 weeks	11.6	11.5	10.5	4.5	6.8^b^	13.5^c^	6.0^c^	42.8^a^
**Bilingualism**								
Yes	13.3	11.0	12.9	4.6	12.7	20.3	7.5	45.2
No	11.7	10.8	9.4^c^	4.7	5.3^c^	12.2^c^	6.0^a^	41.2^b^
**Socioeconomic factors**								
**Disadvantaged area (ZEP)**								
Yes	16.0	11.0	14.2	4.1	16.5	24.5	10.3	47.8
No	11.9^c^	10.9	9.8^c^	4.7	5.9^c^	12.8^c^	6.1^c^	40.4^c^
**Single Parent**								
Yes	15.0	13.4	11.5	6.0	11.6	21.8	9.4	51.7
No	12.0^b^	11.4	10.3	4.4^a^	6.7^c^	13.7^c^	6.2^c^	42.9^c^
**Unemployed father and/or mother**								
Yes	13.6	14.0	13.1	5.3	13.7	23.0	10.6	52.5
No	11.5^b^	10.4^c^	9.1^c^	4.3	3.7^c^	9.9^c^	4.5^c^	39.4^c^
**Occupational category of employed parents**								
Higher-level	9.5	9.6	7.6	4.6	3.3	6.1	3.4	35.0
Intermediate	10.6	11.4	9.0	4.4	3.1	10.3	5.0	37.5
Self-employed	12.1	12.0	11.3	2.5	6.5	13.5	7.2	44.5
Clerical/sales	14.0	11.2	11.0	5.5	7.4	16.2	7.3	47.7
Manual worker	13.6^c^	14.5^b^	15.6^c^	4.9^b^	17.3^c^	28.9^c^	11.2^c^	54.3^c^

*p* respectively ^a^<0,05; ^b^<0,01; ^c^<0,001 for comparison between yes and no for each category of clinical characteristics and socioeconomic factors.

^*^ Children not previously wearing glasses or eye patch.

**Table 3:** Associations between impairments detected in children aged 3 to 4 and socioeconomic factors in the subgroup of children without missing values on adjustment variables.

	Physical impairment				Language impairment n^*^ = 6,245 to 6,836	Psychomotor impairment n^*^ = 6,245 to 6,997			
Socioeconomic factors	Visual Impairment n^*^ = 5,735 to 6,266	Hearing impairment n^*^ = 6,399 to 6,997	Obesity/overweight n^*^ = 6,293 to 6,839	Untreated caries n^*^ = 5,422 to 5,915		Overall motricity	Gross motricity	Fine motricity	Perceptual organization
**Disadvantaged area (ZEP)**									
No	Reference category								
Yes	1.3 [1.1–1.7]	1.1 [0.8–1.3]	1.5 [1.2–1.8]	2.5 [2.0–3.2]	1.8 [1.5–2.2]	1.7 [1.3–2.3]	1.6 [1.0–2.2]	1.8 [1.3–2.7]	2.2 [1.5–3.1]
**Single Parent**									
No	Reference category								
Yes	1.3 [1.0–1.6]	1.3 [1.0–1.6]	1.3 [1.0–1.6]	1.7 [1.2–2.2]	1.8 [1.4–2.2]	1.6 [1.2–2.1]	1.5 [1.0–2.1]	1.7 [1.1–2.6]	2.1 [1.5–3.2]
**Unemployed Father and/or mother**									
No	Reference category								
Yes	1.2 [1.0–1.4]	1.4 [1.2–1.7]	1.4 [1.2–1.7]	3.4 [2.7–4.2]	2.3 [2.0–2.7]	2.4 [1.9–2.9]	1.9 [1.4–2.5]	1.7 [1.2–2.3]	3.4 [2.5–4.6]
**Occupational category of employed parents**									
Higher-level occupations	Reference category								
Intermediate occupations	1.2 [0.9–1.5]	1.2 [1.0–1.6]	1.2 [0.9–1.6]	0.9 [0.5–1.4]	1.8 [1.3–2.4]	1.4 [1.0–2.0]	1.2 [0.7–1.9]	1.4 [0.8–2.4]	2.3 [1.2–4.6]
Self-employed	1.3 [1.0–1.8]	1.3 [1.0–1.7]	1.5 [1.1–2.0]	1.9 [1.2–2.9]	2.4 [1.8–3.2]	2.2 [1.5–3.3]	1.6 [1.0–2.7]	2.6 [1.5–4.5]	3.6 [1.8–7.2]
Clerical/sales worker	1.6 [1.3–2.0]	1.2 [1.0–1.5]	1.6 [1.2–1.9]	2.2 [1.6–3.1]	3.0 [2.3–3.7]	2.1 [1.5–2.8]	1.4 [0.9–2.1]	1.7 [1.1–2.8]	3.9 [2.2–6.9]
Manual worker	1.5 [1.1–2.0]	1.7 [1.3–2.2]	2.1 [1.6–2.8]	5.1 [3.6–7.4]	5.7 [4.3–7.5]	3.4 [2.4–5.0]	2.5 [1.6–4.1]	2.8 [1.6–4.9]	8.8 [4.8–16.3]

Values are odds ratios (95% confidence intervals) from multivariate logistic regression models. Models are adjusted for age, sex, preterm, bilingualism. Additional adjustment for hearing impairment was made for language impairment.

ZEP, Zone d’éducation prioritaire.

*Number of children in the analysis for each screening varies due to missing values in sociodemographic variables.

#### Hearing screening

Hearing screening was performed on 9,521 children (95.8%), and hearing impairments were detected in 10.9% of them (Table 2). Prevalence of hearing impairments did not differ greatly by socioeconomic status and was only moderately higher in children with parents who were unemployed or manual workers (Table 3).

#### Overweight/obesity and thinness screening

BMI was assessed in 8,628 children (87%) and 10.4% were classified as overweight (including obesity) and 2.2% as obese (Table 2). Risk factors for overweight in the multivariate analyses were schooling in a ZEP, having an unemployed parent, and a manual worker parental OC. Children of manual workers were twice as likely to be categorized as overweight as children of parents with a higher-level occupation with comparable clinical characteristics (Table 3).

Among screened children, 397 (4.6%) were thin. The frequency of thinness was higher in children living with a single parent (6.0% vs. 4.4%, *p* = 0.04) (Table 2).

#### Dental screening

Treated caries and at least one untreated carie were found in 2% and 7.3% of the 7,271 screened children (73%), respectively. All socioeconomic criteria were associated with untreated caries in the univariate and multivariate analyses. Untreated caries was the factor most strongly associated with schooling in a ZEP and single parenthood (Tables 2, 3).

#### Speech and language impairment screening

SLI was identified in 14.2% of the 9,325 screened children (93.8%) (Table 2). Risk factors associated with SLI in the multivariate analyses included schooling in a ZEP, single parents, unemployed parents, and lower parental OCs (Table 3). Whatever the socioeconomic factor analyzed, hearing loss was independently associated with SLI [OR 3.5 (2.9–4.2)].

#### Psychomotor impairment screening

Psychomotor development was assessed in 8,960 children (90.1%) and was abnormal in 6.6% of them (Table 2). Gross motricity disability was found in 303 children (3.5%), fine motricity or praxic disorders in 248 children (2.8%) and perceptual disorders in 255 children (2.9%). All socioeconomic variables were significantly associated with overall pyschomotor impairment. In the multivariate analyses, among psychomotor traits screened, perceptual impairments had the strongest gradient based on all socioeconomic factors. In contrast, gross motricity impairments was associated with only a few socioeconomic conditions (unemployment or manual worker parental OC) (Table 3).

#### Vaccination coverage

Data on vaccination coverage were available for 8,627 children (86.7%). DTP and Pertussis vaccination coverage was almost 95% for ≥3 doses. Coverage was 81.2% for 2 MMR doses; 83.1% for ≥3 pneumococcal doses; 61.6% for ≥3 hepatitis B doses; and 69.9% for one dose of meningococcal C after the age of one year. In univariate and multivariate analyses, coverage for DTP, pertussis, MMR was lower in disadvantaged socioeconomic groups, except in schools in ZEPs. Coverage for hepatitis B and pneumococcal doses was even higher in schools in ZEPs than in other schools.

#### Sensitivity analysis

As we had a significant number of missing values for some of the sociodemographic characteristics (Table 1), we re-ran the analysis on the whole sample with a missing value category for each covariate (Supplementary Table). For the two variables with the highest percentage of missing values (unemployment and parental OC) and for the screening result with the strongest social gradient, the OR for the missing category is intermediate between the reference category and the most disadvantaged category.

#### Referrals to health professionals

At the end of PHCS, 4,109 children (41.3%) were referred to at least one practitioner: an ophthalmologist (16.5%), a general practitioner (14.5%), a speech therapist (10.5%), a dentist (8%), an otorhinolaryngologist (7.5%), a psychologist (3.6%), a psychomotrician (0.9%) or a multidisciplinary team (2.6%). Overall, children with lower socioeconomic status were referred more frequently (Table 2).

## Discussion

### Main findings and interpretation

The first aim of the Elfe-PMI study conducted between 2014 and 2016 was to describe the results of the PCHS for children living in 30 French *départements*. A significant proportion of disorders were detected and the study highlights difference by socioeconomic characteristics of preschool children. Since 2014 and even more recently, pediatrics in France has been deteriorating and an increase in disorders and inequalities found in our results is expected. A French national project was launched in December 2022 and allows all child health professionals, as well as school medicine and maternal and child health services, but also parents and children themselves, to contribute to improving a child health system that has become deficient. In order to change the current crisis, it is necessary to have previous comparisons to understand this adverse development and to decide how to proceed.

The large proportion of screened children found to have sensorial disorders, and the differences linked to socioeconomic status demonstrate the utility of this screening (14, 15). These findings may reflect both differences in understanding instructions and real impairments not previously screened for among disadvantaged children. The latter hypothesis is supported by the smaller proportion of children who wore glasses or had seen an ophthalmologist prior to the PCHS in ZEP schools. In systematic review of vision screening in preschool children, vision disorder prevalences ranged from 1% to 81%. Our findings are more similar to the general population (from 1% to 8%) (16).

Childhood hearing loss affects nearly one in five children by age 18. The link between hearing and socioeconomic situation is less clear, and is not always found nor well explained (17). Early identification and intervention of childhood hearing loss may reduce early childhood disadvantages. A recent study found that screening five-year-olds for hearing loss is likely to be cost-effective (18).

The prevalences of overweight/obesity and thinness found in the PCHS are in accordance with other French or European research (19), as are the inequalities linked to social disadvantage (20, 21). A national nursery school survey performed in 2012–3 in France among 5-year-old children reported a slightly higher prevalence of overweight (11.9%), including obesity (3.5%), than in our study, but consistent with the average one year age difference and the declining trend in obesity prevalence observed in France since 2,000 (7).

In the same survey, the prevalence of untreated caries was 10.3%, a level which is also higher than in our study probably due to the age difference. Dental screening in schools is not exhaustive and coverage is clearly inadequate given the prevalence of untreated caries and the large number of referrals to a dentist (the 4th reason for referrals at the end of the PCHS).

Anestimated 14.2% of screened children need close monitoring or treatment for SLI. The prevalence of SLI varies according to the definition, the cut-off point and age, and may range from 3.4% (22) to 17.2% at age 4 years (23) in the literature. Among child-related factors, our results are consistent with the frequently described individual risk factors for SLI such as sex and prematurity. Regarding parental factors, low maternal education is often described as the most predictive factor of SLI (24), a finding in accordance with our results on OC and unemployment.

The prevalence of psychomotor disorders varies according to skill type and socioeconomic indicators. Except in pathological conditions, children's motor acquisition is multidimensional and impacted by their social environment (25). Our results show that assessment of fine motor and perception disorders calls for special attention to children from disadvantaged environments. National recommendations were issued in 2020 to target neurodevelopmental disorders more specifically, but this was not the case at the time of this study (26).

Vaccination coverage according to socioeconomic indicators shows contrasting results. Except for hepatitis B and pneumococcus, vaccination coverage was lower in more disadvantaged children. This was not the case for schools in ZEPs, and hepatitis B and pneumococcal vaccine rates were even higher in ZEPs as previously described (7). This may be related to the fact that PMI services, which provide medical care to a large proportion of disadvantaged families in ZEPs, have an active vaccination policy. Our results for vaccination coverage are comparable to levels observed at national and departmental levels during the study period among children in the third year of nursery school who are one year older than those in our study (27).

Overall, almost one in two children screened in the PCHS were referred to a specialist or a general practitioner. This high proportion points to the existence of a deficit in ambulatory medical follow-up, particularly 2 years after the end of the main vaccination schedule. The tasks of the PMI are not limited to screening children in the context of school health. The PMI provides *pediatric preventive child care from prenatal period through age 6* years and offers the possibility to address the early development of the child. Children are seen at the primary care settings for well-child visits that focus on physical health and development, such as growth and immunisations, but also on monitoring the child's social and emotional development. An advance planning allows the family to schedule the child's medical visits but also allows PMI ‘s team to follow the physician's recommendations. The main objective is to follow up children until the return from the specialist and to ensure that physician's recommandation have been completed or having the school check that the child's needs have been met.

The effectiveness of the PCHS in improving child health and development and narrowing socioeconomic disparities has not been formally assessed, however. Some early and targeted interventions have been shown to be effective, but the efficacy of large-scale screening for vision (14), hearing (28), BMI (29), dental health (30), language (31) and motor (32) disorders is debated in the literature, especially if there is no guarantee of easy access to health services for the families and children most in need. Nevertheless, as preschool education is compulsory in France, schools are a key access point for integrated general health checks that reduce socioeconomic inequities in access to health screening.

Implementation of the national PCHS program at local level is difficult in some places. The resources allocated to preventive primary health care for children are limited and adaptations are needed. First, current changes in the role of childcare nurses may facilitate the implementation of the PCHS through doctor/nurse cooperation protocols. The contribution of education professionals in accordance with ethical rules could also be more formalized. This needs to be properly assessed, however, and can only work through close collaboration between education professionals and primary care professionals (33, 34). A targeted medical examination for children detected by initial screening could be proposed based on “proportionate universalism” (35). Second, the resources allocated to PCHS are not decided at national level but at *départment* level, leading to inequalities in implementation that are not driven by children's needs (6). Standardized national preschool screening guidelines associated with a set of local indicators would guarantee local autonomy and ensure that national public health objectives for young children are met. Third, once identified, child follow-up, which may largely depend on socioeconomic factors, should also be harmonized. Last, national standardization would facilitate the development of protocols that optimize the efficacy of screening tests and would contribute to more accurate assessment of PCHS effectiveness. A French government report summarizes these proposals for improving the PCHS (36). In addition, access to health care services should be facilitated in case of referral of families and children most in need. Currently, speech language pathologist access is very difficult and may not be completed for more than a year. After the vision screening, the orthoptist's visit should be scheduled to expedite the referral to the ophthalmologist. Then it should probably be necessary to provide financial coverage and to deliver suitable packages of support targeting specific vulnerable families to reduce inequalities. Too many families due to lack of support, information and guidance are not involved and not able to take up child health care.

These potential difficulties of implementation and the inconclusive results on the effectiveness of large-scale screening raise the question of the utility of the PCHS. A systematic review of the literature confirms the lack of evidence-based data on the implementation and effectiveness of school health programs (37). However, the authors emphasize that no review has evaluated a multi-component school health intervention such as PCHS that addresses multiple areas of child health.

### Strengths and limitations

One of the strengths of PCHS is that it provides a comprehensive approach to child health. The PCHS is not limited to the content described here, but also assesses children's well-being in ways that are not standardized and measurable in this study and that are best performed in the parents' presence.

**Figure 1:**
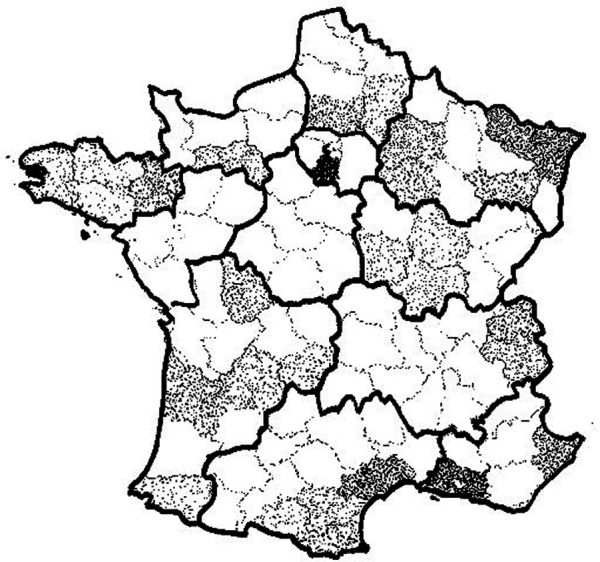
Participating *départements* and density of children born in 2011 and enrolled in school during survey (2014–2016). Each plot represent a child born in 2011 attending nursery schools in the 30 participating *départements* during survey (2014–2016). Aisne (02), Alpes-Maritimes (06), Ariège (09), Aube (10), Aude (11), Bouches-du-Rhône (13), Corrèze (19), Côte-d'Or (21), Côtes-d'Armor (22), Dordogne (24), Finistère (29), Gironde (33), Hérault (34), Ille-et-Vilaine (35), Marne (51), Morbihan (56), Moselle (57), Nièvre (58), Oise (60), Orne (61), Pyrénées-Atlantiques (64), Pyrénées orientales (66), Bas-Rhin (67), Saône-et-Loire (71), Savoie (73), Haute-Savoie (74), Vienne (86), Vosges (88,) Essonne (91), Hauts-de-Seine (92).

This original study, based on the Elfe cohort and resulting from cooperation between research and primary health care services, has some limitations. Although not all French *départements* are represented, the 30 involved represent 33% of the total French population of children below age 5 years in 2015 and cover a large variety of environments ranging from rural to urban (Figure 1). Compared to a random sample of nursery schools in a French 2012–3 survey, the distribution of OCs in our study is shifted towards a larger proportion of higher-level OCs (25% vs. 20%) whereas the proportion of manual workers is similar (10% vs. 12%) (7). This could be explained by a bias linked to participation in the study. The proportion of schools in a ZEP is 10.3% nationally and 9.1% in the 30 participating *départements*, compared to 12.4% in our study, suggesting that the PMI services gave preference to these schools for conducting the PHCS in a context of limited resources. Another limitation is the number of missing values for some covariates that can be explained by the parents' absence when the child was examined. The sensitivity analyses including a missing category show a probability of impairment intermediate between the reference and the most disadvantaged category, and this is consistent with an expected overrepresentation of more disadvantaged situations in cases of missing values.

## Conclusion

PCHS is a comprehensive program aiming to promote the health of preschool children in France carried out by the Maternal and Child Protection services (PMI). The prevalences of impairments observed in our study, which are higher in children from disadvantaged social groups, highlight the potential preventive impact of systematic screening. These results are important to quantify early socioeconomic inequalities in a Western country known for its generous social welfare system. The preschool health screening program in France did not meet the national objective of universality. An harmonized national program with standardized and evaluated content and a coordinated approach should achieve widespread screening based on proportionate universalism and would ensure the reduction of health inequalities with equitable access to screening for all 4-year-olds. Local cooperation between primary health care services, school nurses, general practitioners and specialists should be carried out together and with families. Access to health services for the most vulnerable children referred to a health professional should be improved. Further studies are needed to confirm that the impairments targeted by this multi-component preschool health program receive appropriate follow-up and that interventions of this kind have a positive impact on the later development and health of children in all social categories.

## Data Availability

The original contributions presented in the study are included in the article/**Supplementary Material**, further inquiries can be directed to the corresponding author/s.
