# Supplementary material for: Child health screening program in French nursery schools: Results and related socioeconomic factors
Source: Front Pediatr. 2023 May 4;11:1167539. doi: 10.3389/fped.2023.1167539 (PMC10192858; doi:10.3389/fped.2023.1167539)
Supplement: Supplementary file 1 [file Table1.docx]

**Supplementary table. Associations between impairment detected in children aged 3 to 4 and socioeconomic factors, complete case analysis**

|  | **Physical impairment** | | | | | | | **Language impairment**  n*=9325 | | **Psychomotor impairment**  n*=8960 | | | | | | | |  |
| --- | --- | --- | --- | --- | --- | --- | --- | --- | --- | --- | --- | --- | --- | --- | --- | --- | --- | --- |
| **Socioeconomic factors** | **Visual**  **Impairment**  n*=9417 | **Hearing impairment**  n*=9521 | | **Obesity/**  **overweight**  n*=8628 | | **Untreated caries**  n*=7271 | |  | | **Overall**  **Motricity** | | **Gross motricity** | | **Fine motricity** | | **Perceptual organization** | |  |
| **Age**** | | | |  | |  | |  | |  | |  | |  | |  | |  |
| < 4 years | Reference category | | | | | | | | | | | | | | | | |  |
| ≥ 4 years | 0.9 [0.8-1.0] | 0.8 [0.7-0.9] | | 1.4 [1.2-1.6] | | 1.4 [1.1-1.7] | | 0.7 [0.6-0.8] | | 0.4 [0.3(0.5] | | 0.5 [0.4-0.6] | | 0.4 [0.3-0.5] | | 0.4 [0.3-0.5] | |  |
| **Gender**** | | | |  | |  | |  | |  | |  | |  | |  | |  |
| Male | Reference category | | | | | | | | | | | | | | | | |  |
| Female | 0.9 [0.8-1.1] | 0.8 [0.7-0.9] | | 1.3 [1.2-1.5] | | 0.8 [0.7-1.0] | | 0.5 [0.5-0.6] | | 0.7 [0.6-0.8] | | 0.8 [0.6-1.0] | | 0.6 [0.4-0.7] | | 0.6 [0.5-0.8] | |  |
| **Preterm** | | | |  | |  | |  | |  | |  | |  | |  | |  |
| ≥ 37 weeks | Reference category | | | | | | | | | | | | | | | | |  |
| < 37 weeks | 1.2 [0.9-1.5] | 1.0 [0.7-1.3] | | 0.9 [0.7-1.2] | | 1.5 [1.1-1.8] | | 1.6 [1.3-2.0] | | 1.8 [1.3-2.5] | | 1.5 [1.0-2.3] | | 2.1 [1.4-3.2] | | 1.9 [1.2-2.8] | |  |
| Missing | 1.3 [1.1-1.5] | | 0.7 [0.6-0.8] | 1.0 [0.8-1.2] | | 1.4 [1.1-1.8] | | 1.2 [1.0-1.4] | | 1.2 [0.9-1.5] | | 1.1 [0.8-1.5] | | 1.0 [0.7-1.5] | | 1.0 [0.7-1.4] | |  |
| **Bilingualism** | | | |  | |  | |  | |  | |  | |  | |  | |  |
| No | Reference category | | | | | | | | | | | | | | | | |  |
| Yes | 1.1 [0.9-1.3] | 1.0 [0.9-1.2] | | 1.4 [1.2-1.6] | | 2.3 [1.9-2.7] | | 1.7 [1.5-2.0] | | 1.1 [0.9-1.4] | | 0.8 [0.6-1.1] | | 1.1 [0.8-1.5] | | 1.6 [1.2-2.1] | |  |
| Missing | 1.0 [0.8-1.3] | 1.3 [1.0-1.6] | | 1.1 [0.8-1.4] | | 1.4 [1.0-2.0] | | 0.9 [0.7-1.1] | | 1.4 [1.0-2.0] | | 1.7 [1.1-2.6] | | 2.2 [1.4-3.4] | | 1.9 [1.2-2.9] | |  |
| **Disadvantaged area (ZEP) §** | | | |  | |  | |  | |  | |  | |  | |  | |  |
| No | Reference category | | | | | | | | | | | | | | | | |  |
| Yes | 1.4 [1.1-1.6] | 1.0 [0.8-1.2] | | 1.4 [1.2-1.7] | | 2.6 [2.1-3.2] | | 2.0 [1.7-2.3] | | 1.7 [1.3-2.1] | | 1.6 [1.2-2.2] | | 1.6 [1.1-2.2] | | 2.0 [1.5-2.7] | |  |
| Missing | 0.7 [0.4-1.1] | 1.1 [0.7-1.7] | | 1.1[0.8-1.7] | | 1.6 [0.9-2.7] | | 0.8 [0.5-1.3] | | 0.7 [0.4-1.4] | | 0.9 [0.4-2.0] | | 0.8 [0.3-1.9] | | 1.0 [0.5-2.3] | |  |
| **Single Parent §** | Reference category | | | | | | | | | | | | | | | | |  |
| No |  |  | |  | |  | |  | |  | |  | |  | |  | |  |
| Yes | 1.3 [1.0-1.6] | 1.2 [1.0-1.5] | | 1.1 [0.9-1.4] | | 1.8 [1.4-2.3] | | 1.8 [1.5-2.1] | | 1.6 [1.2-2.1] | | 1.4 [1.0-2.0] | | 1.6 [1.1-2.3] | | 2.0 [1.4-2.9] | |  |
| Missing | 0.9 [0.7-1.1] | 0.6 [0.4-0.7] | | 0.9 [0.7-1.2] | | 1.4 [1.0-1.9] | | 1.0 [0.8-1.2] | | 1.0 [0.7-1.4] | | 0.9 [0.6-1.4] | | 0.5 [0.3-0.9] | | 0.9 [0.5-1.4] | |  |
| **Unemployed Father and/or mother §** | |  | |  | |  | |  | |  | |  | |  | |  | |  |
| No | Reference category | | | | | | | | | | | | | | | | |  |
| Yes | 1.2 [1.0-1.4] | 1.4 [1.2-1.7] | | 1.4 [1.2-1.6] | | 3.5 [2.8-4.3] | | 2.5 [2.2-2.9] | | 2.5 [2.1-3.1] | | 2.1 [1.6-2.7] | | 1.9 [1.4-2.5] | | 3.5 [2.6-4.6] | |  |
| Missing | 1.0 [0.8-1.2] | 0.7 [0.6-0.9] | | 1.0 [0.8-1.3] | | 2.3 [1.7-3.1] | | 1.6 [1.3-1.9] | | 1.4 [1.1-1.9] | | 1.2 [0.8-1.8] | | 0.8 [0.5-1.3] | | 1.3 [0.8-2.0] | |  |
| **Occupational category of employed parents §** | | | | |  | |  | |  | |  | |  | |  | |  | |
| Higher-level occupations | Reference category | | | | | | | | | | | | | | | | |  |
| Intermediate occupations | 1.1 [0.9-1.5] | 1.3 [1.0-1.6] | | 1.2 [0.9-1.5] | | 0.9 [0.6-1.4] | | 1.8 [1.4-2.4] | | 1.3 [0.9-1.9] | | 1.4 [0.9-2.2] | | 1.7 [1.0-2.9] | | 2.7 [1.4-5.1] | |  |
| Self-employed | 1.3 [1.0-1.7] | 1.3 [1.0-1.7] | | 1.5 [1.1-2.0] | | 1.9 [1.3-2.8] | | 2.3 [1.7-3.1] | | 2.3 [1.6-3.4] | | 1.4 [0.9-2.3] | | 2.9 [1.7-4.8] | | 4.0 [2.1-7.9] | |  |
| Clerical/sales worker | 1.5 [1.3-2.0] | 1.2 [1.0-1.5] | | 1.5 [1.2-1.8] | | 2.2 [1.6-3.0] | | 3.1 [2.5-3.9] | | 2.3 [1.7-3.1] | | 1.6 [1.1-2.4] | | 2.1 [1.4-3.3] | | 4.1 [2.4-7.2] | |  |
| Manual worker | 1.5 [1.1-2.0] | 1.6 [1.3-2.1] | | 2.1 [1.6-2.8] | | 5.2 [3.7-7.4] | | 6.0 [4.7-7.8] | | 3.7 [2.6-5.3] | | 2.9 [1.9-4.5] | | 3.4[2.0-5.7] | | 9.4 [5.2-17.0] | |  |
| Missing | 1.4 [1.1-1.8] | 1.1 [0.9-1.4] | | 1.5 [1.1-1.9] | | 4.1 [3.0-5.8] | | 3.6 [2.8-4.7] | | 2.7 [1.9-3.8] | | 1.9 [1.2-3.0] | | 2.0 [1.2-3.3] | | 4.4 [2.4-8.0] | |  |

Values are odds ratios (95% confidence intervals) from multivariate logistic regression models

* Number of screened children

** No missing value

**§** Models are adjusted for age, sex, preterm, bilingualism. Additional adjustement hearing impairment was made for language impairment

ZEP: *Zone d’éducation prioritaire*
